# Distance-Based Estimation Methods for Models for Discrete and Mixed-Scale Data

**DOI:** 10.3390/e23010107

**Published:** 2021-01-14

**Authors:** Elisavet M. Sofikitou, Ray Liu, Huipei Wang, Marianthi Markatou

**Affiliations:** 1 Department of Biostatistics, University at Buffalo, Buffalo, NY 14214, USA; esofikit@buffalo.edu (E.M.S.); huipeiwa@buffalo.edu (H.W.); 2 Head of Oncology Data Science, AstraZeneca PLC, Gaithersburg, MD 20878, USA; ray.liu1@astrazeneca.com

**Keywords:** contingency tables, disparity, mixed-scale data, pearson residuals, residual adjustment function, robustness, statistical distances

## Abstract

Pearson residuals aid the task of identifying model misspecification because they compare the estimated, using data, model with the model assumed under the null hypothesis. We present different formulations of the Pearson residual system that account for the measurement scale of the data and study their properties. We further concentrate on the case of mixed-scale data, that is, data measured in both categorical and interval scale. We study the asymptotic properties and the robustness of minimum disparity estimators obtained in the case of mixed-scale data and exemplify the performance of the methods via simulation.

## 1. Introduction

Minimum disparity estimation has been studied extensively in models where the scale of the data is either interval or ratio (Beran [1], Basu and Lindsay [2]). It has also been studied in the discrete outcomes case. Specifically, when the response variable is discrete and the explanatory variables are continuous, Pardo et al. [3] introduced a general class of distance estimators based on ϕ-divergence measures, the minimum ϕ-divergence estimators, and they studied their asymptotic properties. The estimators can be viewed as an extension/generalization of the Maximum Likelihood Estimator (MLE). Pardo et al. [4] used the minimum ϕ-divergence estimator in a ϕ-divergence statistic to perform goodness-of-fit tests in logistic regression models, while Pardo and Pardo [5] extended the previous works to address solving problems for testing in generalized linear models with binary scale data.

The case where data are measured on discrete scale (either on ordinal or generally categorical scale) has also attracted the interest of other researchers. For instance, Simpson [6] demonstrated that minimum Hellinger distance estimators fulfill desirable robustness properties and for this reason can be effective in the analysis of count data prone to outliers. Simpson [7] also suggested tests based on the minimum Hellinger distance for parametric inference which are robust as the density of the (parametric) model can be nonparametrically estimated. In contrast, Markatou et al. [8] used weighted likelihood equations to obtain efficient and robust estimators in discrete probability models and applied their methods to logistic regression, whereas Basu and Basu [9] considered robust penalized minimum disparity estimators for multinomial models with good small sample efficiency.

Moreover, Gupta et al. [10], Martín and Pardo [11] and Castilla et al. [12] used the minimum ϕ-divergence estimator to provide solution to testing problems in polytomous regression models. Working in a similar fashion, Martín and Pardo [13] studied the properties of the family of ϕ-divergence estimators for log-linear models with linear constraints under multinomial sampling in order to identify potential associations between various variables in multi-way contingency tables. Pardo and Martín [14] presented an overview of works associated with contigency tables of symmetric structure on the basis of minimum ϕ-divergence estimators and minimum ϕ-divergence test statistics. Additional works include Pardo and Pardo [15] and Pardo et al. [16]. Alternative power divergence measures have been introduced by Basu et al. [17].

The class of *f* or ϕ−divergences was originally introduced by Csiszár [18]. The structural characteristics of this class and their relationship to the concepts of efficiency and robustness were studied, for the case of discrete probability models, by Lindsay [19]. Basu and Lindsay [2] studied the properties of estimators derived by minimizing f−divergences between continuous models and presented examples showing the robustness results of these estimates. We also note that Tamura and Boos [20] studied the minimum Hellinger distance estimation for multivariate location and covariance. Additionally, formal robustness results were presented in Markatou et al. [8,21] in connection with the introduction of weighted likelihood estimation.

If *G* is a real valued, convex function, defined on [0,∞) and such that G(u) converges to 0 as u→∞, 0G(0/0)=0, $0G\left(u/0\right)=uG_{\infty }$, $G_{\infty }=\lim_{u\to \infty }\left(G\left(u\right)/u\right)$, the class of ϕ−divergences is defined as

$$\rho \left(\tau ,m_{\beta_{0}}\right)=\sum G\left(\frac{\tau (t)}{m_{\beta_{0}}\left(t\right)}\right)m_{\beta_{0}}\left(t\right),$$

where τ(·), $m_{\beta_{0}}\left(\cdot \right)$ are two probability models. Notice that we define $\rho (\tau ,m_{\beta_{0}})$ on discrete probability models first, where T={0,1,2,⋯,T} is a discrete sample space, *T* possibly infinite, and $m_{\beta_{0}}\left(t\right)\in M=\left\{m_{\beta }\left(t\right):\beta \in B\right\}$, B is the parameter space $B\subseteq R^{d}$. Furthermore, different forms of the function G(u) provide different statistical distances or divergences.

We can change the argument of the function *G* from $\frac{\tau (t)}{m_{\beta_{0}}\left(t\right)}$ to $\frac{\tau (t)}{m_{\beta_{0}}\left(t\right)}-1$. Then, *G* is a function of the Pearson residual which is defined as $\delta \left(t\right)=\frac{\tau (t)}{m_{\beta_{0}}\left(t\right)}-1$, and takes values in [−1,∞). If the measurement scale is interval/ratio, then the Pearson residuals are modified to reflect and adjust for the discrepancy of scale between data, that are always discrete, and the assumed continuous probability model (see Basu and Lindsay [2]).

The Pearson residual is used by Lindsay [19], Basu and Lindsay [2] and Markatou et al. [8,21] in investigating the robustness of the minimum disparity and weighted likelihood estimators, respectively. This residual system allows one to identify distributional errors. If, in the equation of Pearson residual, we replace τ(t) with its best nonparametric representative d(t), the proportion of observations in a sample with value *t*, then $\delta \left(t\right)=\frac{d(t)}{m_{\beta_{0}}\left(t\right)}-1$. We note that the Pearson residuals are called so because $n\sum \delta^{2}\left(t\right)m\left(t\right)$ is Pearson’s chi-squared distance. Furthermore, these residuals are not symmetric since they take values in [−1,∞] and are not standardized to have identical variances.

How does robustness fit into this picture? In the robustness literature, there is a denial of the model’s truth. Following this logic, the framework based on disparities starts with goodness-of-fit by identifying a measure that assesses whether the model fits the data adequately. Then, we examine whether this measure of adequacy is robust and in what sense. A fundamental tool that assists in measuring the degree of robustness is the Pearson residual, because it measures model misspecification. That is, Pearson residuals provide information about the degree to which the specified model $m_{\beta }$ fits the data. In this context, outliers are defined as those data points that have a low probability of occurrence under the hypothesized model. Such probabilistic outliers are called *surprising observations* (Lindsay [19]). Furthermore, the robustness of estimators obtained via minimization of the divergence measures we discuss here is indicated by the shape of the associated Residual Adjustment Function (RAF), a concept that is reviewed in Section 2. Of note is that in contingency table analysis, the generalized residual system is used for examination of sources of error in models for contingency tables, see, for example, Haberman [22], Haberman and Sinharay [23]. The concept of generalized residuals in the case of generalized linear models is discussed, for example, in Pierce and Schafer [24].

Data sets are comprised of data measured on both categorical (ordinal or nominal) scale and interval/ratio scale. We can think of these data as realizations of discrete and continuous random variables respectively. Examples of data sets that include mixed-scale data are electronic health records containing diagnostic codes (discrete) and laboratory measurements (e.g., blood pressure, alanine amino transferase (ALT) measurements on interval/ratio scale) and marketing data (customer records include income and gender information). Additional examples include data from developmental toxicology (Aerts et al. [25]), where fetal data from laboratory animals include binary, categorical and continuous outcomes. In this context, the joint density of the discrete and continuous random variables is given as $m_{\beta }\left(x,y\right)=f_{\beta_{1}}\left(y|x\right)g_{\beta_{2}}\left(x\right)$, where $\beta^{T}=\left(\beta_{1}^{T},\beta_{2}^{T}\right)$ are parameter vectors indexing the joint, conditional on *x* and probability density function of *x*.

Work on the analysis of mixed-scale data is complicated by the fact that is difficult to identify suitable joint probability distributions to describe both measurement scales of the data, although a number of ad hoc methods to the analysis of mixed-scale data have been used in applications. Olkin and Tate [26] proposed multivariate correlation models for mixed-scale data. Copulas also provide an attractive approach to modeling the joint distribution of mixed-scale data, though copulas are less straightforward to implement, and there are subtle identifiability issues that complicate the specification of a model (Genest and Nešlehová [27]).

To formulate the joint distribution in the mixed-scale variables case one can either specify the marginal distribution of the discrete variables and the conditional distribution of the continuous variables. Alternatively, one can specify the marginal distribution of the continuous variables and the conditional distribution of the discrete variables given the continuous variables. Of note here is that the direction of factorization generally yields distinct model interpretations and results. The first approach has received much attention in the literature, in the context of the analysis of data with mixtures of categorical and continuous variables. Here, the continuous variables follow different multivariate normal distributions for each possible setting of the categorical variable values; the categorical variables then follow an arbitrary marginal multinomial distribution. This model is known in the literature as the conditional Gaussian distribution model and is central in the discussion of graphical association models with mixed-scale variables (Lauritzen and Wermuth [28]). A very special case of this model is used in our simulations.

In this paper, we develop robust methods for mixed-scale data. Specifically, Section 2 reviews basic concepts in minimum disparity estimation, Section 3 defines Pearson residuals for data measured in discrete, interval/ratio and mixed-scale, and studies their properties. Section 4 establishes the optimization problem for obtaining estimators of the model parameters, while Section 5 and Section 6 establish the robustness and asymptotic properties of these estimators. Finally, Section 7 presents simulations showing the performance of these methods and Section 8 offers discussions. The Appendix A includes proofs of the theoretical results.

## 2. Concepts in Minimum Disparity Estimation

Beran [1] introduced a robust method to estimate the parameters of a statistical model, called minimum Hellinger distance estimation. The parameter estimator is obtained by minimizing the Hellinger distance between a parametric model density and a nonparametric density estimator. Lindsay [19] extended the aforementioned method to incorporate many other distances, and introduced the concept of the residual adjustment function in the context of minimum disparity estimation. The Minimum Distance Estimators (MDE) of a parameter vector β are obtained by minimizing over β, the distance (or disparity)

(1) $$\rho \left(d,m_{\beta }\right)=\sum_{x}G\left(\delta \left(x\right)\right)m_{\beta }\left(x\right),$$

where the assumed model $m_{\beta }$ is a probability mass function. When the model $m_{\beta }$ is continuous, the MDE of the parameter vector β is obtained by minimizing over β the quantity

(2) $$\rho \left(f^{*},m_{\beta }^{*}\right)=\int G\left(\delta \left(x\right)\right)m_{\beta }^{*}\left(x\right)\;dx,$$

where $f^{*}\left(x\right)=\int k\left(x;t,h\right)d\hat{F}\left(t\right)$, $m_{\beta }^{*}\left(x\right)=\int k\left(x;t,h\right)m_{\beta }\left(t\right)\;dt$, $\hat{F}$ is the empirical distribution function obtained from the data and *k* is a smooth family of kernel functions. One example is the normal density with mean *t* and standard deviation *h*. Furthermore, δ(x) is the Pearson residual defined as $\delta \left(x\right)=f^{*}\left(x\right)/m^{*}\left(x\right)-1$. Lindsay [19] and Basu and Lindsay [2] discuss the efficiency and robustness properties of these estimators.

If $G\left(\delta \right)=\frac{1}{\lambda (1+\lambda )}\left\{{\left(1+\delta \right)}^{\left(\lambda +1\right)}-1\right\}$ we obtain the class of power divergence measures. Notice that we have G(0)=0. Different values of λ offer different measures; for example, when λ=−2 we obtain Neyman’s chi-squared divided by 2 measure, while λ=−1,−1/2 return the Kullback-Leibler and Hellinger distances, respectively.

Under appropriate conditions, (1) and (2) can be written as

$$\sum A\left(\delta \left(x\right)\right)m_{\beta }\left(x\right)=0,$$

or

$$\int A\left(\delta \left(x\right)\right)\nabla m_{\beta }^{*}\left(x\right)\;dx=0,$$

where $A\left(\delta \right)=\left(\delta +1\right)G^{\prime }\left(\delta \right)-G\left(\delta \right)$ and the prime denotes differentiation with respect to δ.

Lindsay [19] has shown that the structural characteristics of the function A(δ) play an important role in the robustness and efficiency properties of these methods. Furthermore, without loss of generality, we can center and rescale A(δ), and define the RAF as follows.

**Definition** **1** (Lindsay [19]). *Let* A(δ) *be an increasing and twice differentiable function on* [−1,∞) *defined as*

$$\begin{array}{cc} A(\delta ) & =\left(\delta +1\right)G^{\prime }\left(\delta \right)-G\left(\delta \right),\\ A(0) & =0,\\ A^{\prime }\left(0\right) & =1, \end{array}$$

*where G is strictly convex and twice differentiable with respect to δ on* [−1,∞) *with* G(0)=0*. Then,* A(δ) *is called residual adjustment function.*

**Remark** **1.** *Since* $A^{\prime }\left(\delta \right)=\left(1+\delta \right)G^{\prime \prime }\left(\delta \right)$ *, the second order differentiability of G, in addition to its strict convexity, implies that* A(δ) *is strictly increasing function of δ on* [−1,∞) *. Thus, we can define* A(δ) *as above without changing the solutions of the aforementioned estimating equations in the discrete case (see Lindsay [19], p. 1089). In the continuous case, such standardization does not change the estimating properties of the associated disparities (see Basu and Lindsay [2], p. 687).*

Two fundamental and at the same time conflicting goals in robust statistics are the goals of robustness and efficiency. In the traditional literature on robustness, first order efficiency is sacrificed and, instead, safety of the estimation or testing method against outliers is guaranteed. Here, one adheres to the notion that information about robustness of a method is carried by the influence function. In our setting, using the influence function to characterize the robustness properties of the associated estimation procedures is misleading. Instead, the shape of the RAF, A(·), provides information to the extent of which our procedures can be characterized as robust. The interested reader is directed to Lindsay [19] for further discussion on this topic.

## 3. Pearson Residual Systems

In this section, we define various Pearson residuals, appropriate for the measurement scale of the data. We introduce our notation first.

Let $(y_{i},x_{i}),\;i=1,2,\cdots ,n$ be realizations from *n* independent and identically distributed random variables that follow a distribution with density $m_{\beta }\left(x,y\right)$. Recall that we use the word density to denote a general probability function, independently of whether the random variables X,Y are discrete, continuous or mixed. In what follows, we define different Pearson residual systems that account for the measurement scale of the data and study their properties.

**Case 1:** *Both X and Y are discrete.*

In this case, the pairs $\left(y_{i},x_{i}\right)$ follow a discrete probability mass function $m_{\beta }\left(x_{i},y_{i}\right)$. Define the Pearson residual as

$$\delta \left(x,y\right)=\frac{\frac{n_{x,y}}{n}}{m_{\beta }\left(y|x\right)\pi_{x}}-1,$$

where $\pi_{x}=P\left(X=x\right)=g\left(x\right)$, and $n_{x,y}$ is the number of observations in the cell with Y=y and X=x.

Note that this definition of the Pearson residual is nonparametric on the discrete support of *X*. In the case of regression, one can carry out a semiparametric argument to obtain the estimators of the vector β and $\pi_{x}$.

We now establish that, under correct model specification, the residual δ(x,y) converges, almost surely, to zero.

**Proposition** **1.** *When the model is correctly specified and as* n→∞ *,*

$$\delta \left(x,y\right)\overset{a.s.}{\to }0.$$

**Proof.**  Write

$$\begin{array}{cc} \delta (x,y) & =\frac{\frac{n_{x,y}}{n}}{m_{\beta }\left(y|x\right)\pi_{x}}-1\\ \; & =\frac{\frac{n_{x,y}}{n_{x}}\cdot \frac{n_{x}}{n}}{m_{\beta }\left(y|x\right)\pi_{x}}-1. \end{array}$$

Then

$$\begin{array}{cc} \frac{n_{x}}{n} & =\frac{(\#\;\mathrm{of}\;\mathrm{observations}\;\mathrm{in}\;\mathrm{the}\;\mathrm{sample}\;\mathrm{equal}\;\mathrm{to}\;x)}{n}\\ \; & =\frac{1}{n}\sum_{i=1}^{n}I\left(x_{i}=x\right), \end{array}$$

where I(·) is the indicator function. Furthermore,

$$E\left[\frac{1}{n}I\left(X_{i}=x\right)\right]=P\left(X=x\right)<\infty ,$$

and by the strong law of large numbers

$$\frac{n_{x}}{n}\overset{a.s.}{\underset{n\to \infty }{\to }}E\left[I\left(X=x\right)\right]=P\left(X=x\right)=\pi_{x}.$$

Similarly,

$$\frac{n_{x,y}}{n_{x}}\overset{a.s.}{\to }m_{\beta }\left(y|x\right),$$

therefore

$$\delta \left(x,y\right)\overset{a.s.}{\underset{n\to \infty }{\to }}0$$

under correct model specification. □

**Case 2:** *Y is continuous and X is discrete.*

This is the case in some *ANOVA* models. We can still define the Pearson residual in this setting as

$$\delta \left(x,y\right)=\frac{f_{n}\left(y,x\right)}{m_{\beta }\left(y,x\right)}-1,$$

where

$$\begin{array}{cc} f_{n}\left(y,x\right) & =f_{n}^{*}\left(y|x\right)g\left(x\right)\\ \; & =\left\{\int k\left(y,t,h\right)\;d{\hat{F}}_{n}\left(t|x\right)\right\}\frac{n_{x}}{n} \end{array}$$

and

$$\begin{array}{cc} m_{\beta }\left(y,x\right) & =m_{\beta }^{*}\left(y|x\right)g\left(x\right)\\ \; & =\left\{\int k\left(y,t,h\right)\;dM_{\beta }\left(t|x\right)\right\}\pi_{x}. \end{array}$$

Then,

$$\delta \left(x,y\right)=\frac{f_{n}^{*}\left(y|X=x\right)\frac{n_{x}}{n}}{m_{\beta }^{*}\left(y|X=x\right)\pi_{x}}-1.$$

**Proposition** **2.** *Assume the model is correctly specified and* k(y,t,h) *is a continuous function. Then,*

$$\delta \left(x,y\right)\overset{a.s.}{\underset{n\to \infty }{\to }}0.$$

**Proof.**  Under the strong law of large numbers

$$\frac{n_{x}}{n}\overset{a.s.}{\underset{n\to \infty }{\to }}\pi_{x}.$$

Under the correct model specification, continuity of the kernel function and the fact that ${\hat{F}}_{n}$ converges completely to *F* (implication of Glivenko-Cantelli theorem),

$$\lim_{n\to \infty }\int k\left(y;t,h\right)\;d{\hat{F}}_{n}\left(t|x\right)\to \int k\left(y;t,h\right)\;dF\left(t|x\right)=\int k\left(y;t,h\right)\;dM_{\beta }\left(t|x\right)=m_{\beta }^{*}\left(y|x\right)$$

(extension of Helly-Bray lemma). Therefore,

$$\frac{\frac{n_{x}}{n}\;f_{n}^{*}\left(y|x\right)}{\pi_{x}\;m_{\beta }^{*}\left(y|x\right)}\overset{a.s.}{\to }\frac{\pi_{x}}{\pi_{x}}\cdot \frac{m_{\beta }^{*}\left(y|x\right)}{m_{\beta }^{*}\left(y|x\right)}=1$$

and hence

$$\delta \left(x,y\right)=\frac{\frac{n_{x}}{n}\;f_{n}^{*}\left(y|x\right)}{\pi_{x}\;m_{\beta }^{*}\left(y|x\right)}-1\overset{a.s.}{\to }1-1=0.$$

 □

**Case 3:** *Y is continuous and X is continuous.*

In this case, the pairs $\left(y_{i},x_{i}\right)$ follow a continuous probability distribution. The Pearson residual is then defined as

$$\delta \left(x,y\right)=\frac{f_{n}^{*}\left(y,x\right)}{m_{\beta }^{*}\left(y,x\right)}-1,$$

where

$$\begin{array}{cc} f_{n}^{*}\left(x,y\right) & =\int k\left(x,y;t_{1},t_{2}\right)\;d{\hat{F}}_{n}\left(t_{1},t_{2}\right),\\ m_{\beta }^{*}\left(x,y\right) & =\int k\left(x,y;t_{1},t_{2}\right)m_{\beta }\left(t_{1},t_{2}\right)\;dt_{1}dt_{2}. \end{array}$$

As an example, we take the linear regression model with random carriers *X*, and $\epsilon_{i}∼N\left(0,1\right)$. Furthermore, assume that the random carriers follow a normal distribution with mean vector μ and covariance matrix Σ. In this case, $y_{i}=x_{i}^{T}\beta +\epsilon_{i}$ and the quantities $z_{i}=\left(y_{i}-x_{i}^{T}\beta \right)/\sigma$ are independent, identically distributed random variables when β represents the vector of true parameters. Hence, the $z_{i}$’s represent realizations of a random variable *Z* that has a completely known density f(z). Thus,

$$m_{\beta }\left(x,y\right)=m_{\beta }\left(z|x\right)\cdot g\left(x\right),\;z=\left(y-x^{T}\beta \right)/\sigma$$

and hence

$$\begin{array}{cc} m_{\beta }^{*}\left(x,y\right) & =m_{\beta }^{*}\left(y-x^{T}\beta |X=x\right)g^{*}\left(x\right),\\ m_{\beta }^{*}\left(y-x^{T}\beta |X=x\right) & =m_{\beta }^{*}\left(z|x\right)=\int k\left(z,t,h\right)\;dM_{\beta }\left(t|x\right),\\ g^{*}\left(x\right) & =\int k^{\prime }\left(x,t^{\prime },h^{\prime }\right)g\left(t^{\prime }\right)\;dt^{\prime }. \end{array}$$

The kernel k(z,t,h) is selected so that it facilitates easy computation. Kernels that do not entail loss of information when they are used to smooth the assumed parametric model are called transparent kernels (Basu and Lindsay [2]). Basu and Lindsay [2] provide a formal definition of transparent kernels and an insightful discussion on the point of why transparent kernels do not exhibit information loss when convoluted with the hypothesized model (see Section 3.1 of Basu and Lindsay [2]).

## 4. Estimating Equations

In this section, we concentrate on cases 1, 2 presented in the previous section. We carefully outline the optimization problems and discuss the associated estimating equations for these two cases. The case where both *X* and *Y* are continuous has been discussed in the literature, see, for example, Markatou et al. [21].

**Case 1:** *Both **X** and **Y** are discrete.*

In this case, the minimum distance estimators of the parameter vector β and $\pi_{x}$ are obtained by solving the following optimization problem

(3) $$\min_{\beta ,\pi_{x}}\;\rho \left(d,m_{\beta }\right)$$

subject to

$$\sum_{x}\pi_{x}=1.$$

Optimization problem (3) is equivalent to the problem

$$\min\;\sum_{x,y}G\left(\delta \left(x,y\right)\right)m_{\beta }\left(x,y\right)$$

subject to

$$\sum_{x}\pi_{x}=1.$$

The class of *G* functions that we use creates distances that belong in the family of ϕ-divergences.

**Proposition** **3.** *The estimating equations for **β** and* $\pi_{x}$ *are given as:*

(4) $$\begin{array}{c} \sum_{x,y}w\left(\delta \left(x,y\right)\right)\;n_{x,y}\;u\left(y|x;\beta \right)=0,\\ \sum_{x,y}w\left(\delta \left(x,y\right)\right)\;n_{x,y}\;\left\{\frac{I(X=x)}{\pi_{x}}-1\right\}=0. \end{array}$$

*The function* w(δ(x,y)) *is a weight function, such that* 0≤w(δ(x,y))≤1 *, and it is defined as*

$$w\left(\delta \left(x,y\right)\right)=\min\left\{\frac{{\left[A\left(\delta \left(x,y\right)\right)+1\right]}^{+}}{\delta (x,y)+1},1\right\}$$

*with* ${\left[\cdot \right]}^{+}$ *indicating the positive part of the function* A(δ(x,y))+1 *.*

**Proof.**  The main steps of the proof are provided in the Appendix A.1. □

**Remark** **2.**

1. *The above two estimating equations can be solved with respect to **β** and* $\pi_{x}$ *. In an iterative algorithm, we can solve the second equation (4) explicitly for* $\pi_{x}$ *to obtain*
  $$\pi_{x}=\frac{\sum_{y}w\left(\delta \left(x,y\right)\right)n_{x,y}}{\sum_{x,y}w\left(\delta \left(x,y\right)\right)n_{x,y}}.$$
  *This means that if the model does not fit any of the y, observed at a particular x well, the weight for this x will drop as well.*
2. *When* A(δ(x,y))=δ(x,y) *the corresponding estimating equation for β becomes* $\sum_{x,y}n_{x,y}u\left(y|x;\beta \right)=0$ *and the MLE is obtained. This is because the corresponding weight function* w(δ(x,y))=1 *. In this case, the estimating equations for the* $\pi_{x}$ *s become* $\sum n_{x,y}\left[\frac{I(X=x)}{\pi_{x}}-1\right]=0$ *, the estimating equations for the MLEs of* $\pi_{x}$ *.*
3. *The Fisher consistency property of the function that introduces the estimates guarantees that the expectation of the corresponding estimating function is 0, under the correct model specification.*

**Case 2:** ***Y** is continuous and **X** is discrete.*

In this case, the estimates of the parameters β and $\pi_{x}$ are obtained by solving the following optimization problem

$$\min_{\beta ,\pi_{x}}\;\sum_{x}\int G\left(\delta \left(x,y\right)\right)m_{\beta }^{*}\left(y,x\right)dy$$

subject to

$$\sum_{x}\pi_{x}=1.$$

In general $m_{\beta }^{*}\left(y,x\right)=m_{\beta }^{*}\left(y|x\right)\pi_{x}$; in the case where y,x are independent $m_{\beta }^{*}\left(y,x\right)=m_{\beta }^{*}\left(y\right)\pi_{x}$, and the optimization problem stated above is equivalent to

(5) $$\min_{\beta ,\pi_{x}}\;\sum_{x}\pi_{x}\int G\left(\delta \left(x,y\right)\right)m_{\beta }^{*}\left(y\right)dy$$

subject to

$$\sum_{x}\pi_{x}=1.$$

**Proposition** **4.** *The estimating equations for β and* $\pi_{x}$ *in the case of independence of* y,x *are given as follows:*

(6) $$\begin{array}{c} \sum_{x}\pi_{x}\int A\left(\delta \left(x,y\right)\right)\nabla_{\beta }\;m_{\beta }^{*}\left(y\right)dy=0,\\ \sum_{x}\pi_{x}\int A\left(\delta \left(x,y\right)\right)\left[\frac{I(X=x)}{\pi_{x}}-1\right]\;m_{\beta }^{*}\left(y\right)dy=0, \end{array}$$

*where* A(δ) *is the residual adjustment function (RAF) that corresponds to the function G, and* $G^{\prime }\left(\delta \right)$ *is the derivative of G with respect to δ.*

**Proof.**  Straightforward, after differentiating the Lagrangian with respect to β and $\pi_{x}$. □

**Case 3:** ***Y** is continuous and **X** is continuous.*

In this case, we refer the reader to Basu and Lindsay [2].

## 5. Robustness Properties

Hampel et al. [29] and Hampel [30,31] define robust statistics as the “statistics of approximate parametric models”, and introduce one of the fundamental tools of robust statistics, the concept of the influence function, in order to investigate the behavior of a statistic $T_{n}$ expressed as a functional T(G). The influence function is a heuristic tool with the intuitive interpretation of measuring the bias caused by an infinitesimal contamination at a point *x* on the estimate standardized by the mass of contamination. Its formal definition is as follows:

**Definition** **2.** *The influence function of a functional T at the distribution F is given as*

$$IF\left(x;T,F\right)=\lim_{t\to 0}\frac{T(\left(1-t\right)F+t\Delta_{x})-T\left(F\right)}{t},$$

*in those* x∈X *where the limit exists,* 0≤t≤1 *and* $\Delta_{x}$ *is the Dirac measure defined as*

(7) $$\Delta_{x}\left(u\right)=\left\{\begin{array}{cc} 1, & u=x,\\ 0, & u\neq x. \end{array}\right.$$

If an estimator has a bounded influence function, the estimator is considered to be robust to outliers, that is data which is away from the pattern set by the majority of the data. The effect of bounding the influence function is the sacrifice of efficiency; estimators with bounded influence function, while are not affected by outlying points, are not fully efficient under the correct model specification.

Our goal in calculating the influence function is to show the full efficiency of the proposed estimators. That is, the influence function of the proposed estimators, under correct model specification, equals the influence function of the corresponding maximum likelihood estimators. In our context, robustness of the estimators is quantified by the associated RAFs (see Lindsay [19] and Basu and Lindsay [2]).

In what follows, we will derive the influence function of the estimators for the parameter vector β in the case where both y,x are discrete. Similar calculations provide the influence functions of estimators obtained under the remaining scenarios. To do so, we need to resort to the estimators’ functional form, denoted by $\beta_{\epsilon }$, with corresponding estimating equations

$$\sum_{s,t}w\left(\delta_{\epsilon }\left(s,t\right)\right)u\left(t|s;\beta_{\epsilon }\right)d_{\epsilon }\left(s,t\right)=0,$$

where $d_{\epsilon }\left(s,t\right)=\left(1-\epsilon \right)d\left(s,t\right)+\epsilon \Delta_{x,y}\left(s,t\right).$ The influence function is then obtained by differentiating the aforementioned estimating equations with respect to ϵ and then evaluating the derivative at ϵ=0.

**Proposition** **5.** *The influence function of the β estimator is given by*

$$\beta_{0}^{\prime }={\left[A\left(d\right)\right]}^{-1}B\left(x,y;d\right),$$

*where*

$$\begin{array}{cc} A(d)= & \sum_{s,t}\left[\delta_{0}\left(t\right)+1\right]w^{\prime }\left(\delta_{0}\left(s,t\right)\right)u\left(t|s;\beta_{0}\right)u^{T}\left(t|s;\beta_{0}\right)d\left(s,t\right)\\ \; & -\sum_{s,t}w\left(\delta_{0}\left(s,t\right)\right)\nabla u\left(t|s;\beta_{0}\right)d\left(s,t\right), \end{array}$$

$$\begin{array}{cc} B(x,y;d)= & \sum_{s,t}\left[\frac{I(s=x,t=y)}{m_{\beta_{0}}\left(t|s\right)\pi_{s}}-\frac{d(s,t)}{m_{\beta_{0}}\left(t|s\right)\pi_{s}}w^{\prime }\left(\delta_{0}\left(s,t\right)\right)\right]u\left(t|s;\beta_{0}\right)d\left(s,t\right)\\ \; & -\sum_{s,t}w\left(\delta_{0}\left(s,t\right)\right)u\left(t|s;\beta_{0}\right)d\left(s,t\right)+w\left(\delta_{0}\left(x,y\right)\right)u\left(t|s;\beta_{0}\right), \end{array}$$

*with* $u\left(t|s;\beta \right)=\nabla \ln m_{\beta }\left(t|s\right)$ *, and the subscript 0 indicates evaluation at a parametric model.*

**Proof.**  The proof is obtained via straightforward differentiation and its main steps are provided in the Appendix A.2. □

**Proposition** **6.** *Under the assumption that the model is correct, the influence function derived, reduces to the influence function of the MLE of **β**.*

**Proof.**  Under the assumption that the adopted model is the correct model, the density d(s,t) is $m_{\beta_{0}}\left(s,t\right)$, so that δ(s,t)=0. Now recall that w(0)=1 and $w^{\prime }\left(0\right)=0$, so the expression A(d) reduces to

(8) $$\begin{array}{cc} A(d) & =-\sum_{s,t}\nabla u\left(t|s;\beta_{0}\right)m_{\beta_{0}}\left(s,t\right)\\ \; & =i(\beta ,x,y). \end{array}$$

Furthermore, the expression B(x,y;d) reduces to $u(y|x;\beta_{0})$, where we assume exchangeability of differentiation and integration and use the fact that $u\left(t|s;\beta_{0}\right)=u\left(s,t;\beta_{0}\right)$. Hence, the influence function is given as

$$i^{-1}\left(\beta ;x,y\right)u\left(y|x;\beta_{0}\right),$$

which is exactly the influence function of the MLE. Therefore, full efficiency is preserved under the model. □

## 6. Asymptotic Properties

In what follows, we establish asymptotic normality of the estimators in the case of discrete variables. The techniques for obtaining asymptotic normality in the mixed-scale case are similar and not presented here. 

**Case 1:** *Both **X** and **Y** are discrete.*

Recall that the k−th estimating equation is given as $\sum_{x,y}w\left(\delta_{\beta }\left(x,y\right)\right)n_{x,y}u_{k}\left(y|x;\beta \right)=0$, which can be expanded in Taylor series in the neighborhood of the true parameter $\beta_{0}$ to obtain:

(9) $$\frac{1}{n}\sum_{x,y}w\left(\delta_{\beta }\left(x,y\right)\right)n_{x,y}u_{k}\left(y|x;\beta \right)≅A_{n}+{\left(\beta -\beta_{0}\right)}^{T}B_{n}+\frac{1}{2}{\left(\beta -\beta_{0}\right)}^{T}C_{n}\left(\beta -\beta_{0}\right),$$

where

(10) $$\begin{array}{cc} A_{n} & =\frac{1}{n}\sum_{x,y}w\left(\delta_{\beta }\left(x,y\right)\right)n_{x,y}u_{k}\left(y|x;\beta_{0}\right),\\ B_{n} & =\nabla_{\beta }\left\{\frac{1}{n}\sum_{x,y}w\left(\delta_{\beta }\left(x,y\right)\right)n_{x,y}u_{k}\left(y|x;\beta \right)\right\}{|}_{\beta_{0}}, \end{array}$$

$C_{n}$ is a p×p Hessian matrix whose (t,e)−th element is given as

$$\frac{\partial^{2}}{\partial \beta_{t}\partial \beta_{e}}\left\{\frac{1}{n}\sum_{x,y}w\left(\delta_{\beta }\left(x,y\right)\right)n_{x,y}u_{k}\left(y|x;\beta \right)\right\}{|}_{\beta_{0}}.$$

Under assumptions 1–8, listed in the Appendix A.3, we have the following theorem.

**Theorem** **1.** *The minimum disparity estimators of the parameter vector **β** are asymptotically normal with asymptotic variance* $I^{-1}\left(\beta_{0}\right)$ *, where* I(·) *indicates the Fisher information matrix.*

## 7. Simulations

The simulation study presented below has two aims. The first one, is to indicate the versatility of the disparity methods for different data measurement scales. The second aim is to exemplify and study the robustness of these methods under different contamination scenarios.

**Case 1:** *Both X and Y are discrete.*

The Cressie-Read family of power divergence is given by

$$PWD\left(d,m_{\beta }\right)=\sum m_{\beta }\left(x,y\right)\cdot \frac{{\left[1+\delta \left(x,y\right)\right]}^{\lambda +1}-1}{\lambda (\lambda +1)}=\sum d\left(x,y\right)\cdot \frac{{\left[d\left(x,y\right)/m_{\beta }\left(x,y\right)\right]}^{\lambda }-1}{\lambda (\lambda +1)},$$

where $d\left(x,y\right)=n_{x,y}/n$ is the proportion of observations with value x,y and $m_{\beta }\left(x,y\right)=m_{\beta }\left(y|x\right)\pi_{x}$ is the density function of the model of interest.

To evaluate the performance of our algorithmic procedure, we use the following disparity measures, that is,

$$\begin{array}{cc} & \mathrm{Likelihood}\mathrm{disparity}\;(\lambda =0):\\ \; & LD\left(d,m_{\beta }\right)=\sum d\left(x,y\right)\cdot \left\{\log[d\left(x,y\right)/m_{\beta }\left(x,y\right)]\right\},\\ & \mathrm{Twice}-\mathrm{squared}{\mathrm{Hellinger}}^{\prime }s\;\left(\lambda =-1/2\right):\\ \; & HD\left(d,m_{\beta }\right)=2\cdot \sum {\left[\sqrt{d(x,y)}-\sqrt{m_{\beta }\left(x,y\right)}\right]}^{2},\\ & \mathrm{Pearson}’s\mathrm{chi}-\mathrm{squared}\mathrm{divided}\mathrm{by}2\;(\lambda =1):\\ \; & PCS\left(d,m_{\beta }\right)=\sum \frac{{\left[d\left(x,y\right)-m_{\beta }\left(x,y\right)\right]}^{2}}{2\cdot m_{\beta }\left(x,y\right)},\\ & \mathrm{Symmetric}\mathrm{chi}-\mathrm{squared}\;\left(G\left(\delta \left(x,y\right)\right)=\frac{2{\left[\delta \left(x,y\right)\right]}^{2}}{\delta (x,y)+2}\right):\\ \; & SCS\left(d,m_{\beta }\right)=2\cdot \sum \frac{{\left[m_{\beta }\left(x,y\right)-d\left(x,y\right)\right]}^{2}}{\left[m_{\beta }\left(x,y\right)+d\left(x,y\right)\right]}. \end{array}$$

The data are generated in four different ways using three different sample sizes *N*, say N=100;N=1000 and N= 10,000. The data format used can be represented in a 5×5 contingency table, with $n_{i,j}$, i=1,2,⋯,5; j=1,2,⋯,5 denoting the counts in the ij-th cell, $n_{i•}$ and $n_{•j}$ representing the row and column totals, respectively. Furthermore, the variable *x* indicates columns, while *y* indicates the rows. In each of the aforementioned cases/scenarios, 10,000 tables were generated and that corresponds to the number of Monte Carlo (MC) replications. Our purpose is to get the mean values of the estimates of the parameters $m_{\beta }\left(y|x\right)$’s and $\pi_{x}$’s along with their corresponding standard deviations (SDs). Notice that, in this setting, the estimation of $\pi_{x}$ and $m_{\beta }\left(y|x\right)$ is completely nonparametric, that is, no model is assumed for estimating the marginal probabilities of *X* and *Y*.

The table was generated by using either a fixed total sample size *N* or fixed marginal probabilities. These two data generating schemes imply two different sampling schemes that could have generated the data with consequences for the probability model one would use. For example, with fixed total sample size the distribution of the counts is multinomial, or if the row margin is fixed in advance the distribution of the counts is a product binomial distribution. In the former case of fixed *N*, we explored two different scenarios: a balanced and an imbalanced one. The imbalanced scenario allows for the presence of one zero cell in the contingency table, whereas the balanced scenario does not. In the latter case of fixed marginal probabilities, the row marginal probabilities ($m_{\beta }\left(y|x\right)$’s) were fixed, while the column marginals ($\pi_{x}$’s) were randomly chosen and these values were used to obtain the contingency table. In this case, we also explored a balanced and an imbalanced scenario based on whether the row marginal probabilities were chosen so that to be equal to each other or not, respectively.

Specifically, under Scenario Ia, where the total sample size *N* was fixed and the balanced design was exploited, none of the $n_{ij}$’s ($n_{ij}\neq 0,\;\forall \;i,j=1,2,3,4,5$) was set equal to zero, with equal row and column marginal probabilities. Table 1 presents the mean of 10,000 estimates and the corresponding SDs for all four distances (PCS,HD,SCS,LD) when *N* is fixed under the balanced scenario. Table 1 clearly shows that all distances provide estimates approximately equal to 0.200 regardless of the sample size used. Furthermore, as the sample size increases, the SDs decrease noticeably.

In Scenario IIa, where the total sample size *N* was fixed and the contingency table was structured using the imbalanced design, the presence of a zero cell ($n_{11}=0$) was allowed. The results of this scenario are presented in Table 2, where the estimates were calculated exploiting all disparity measures. For the LD, $n_{11}$ was set equal to $10^{-8}$. The presence of zero cells in contingency tables has a large history in the relevant literature on contingency tables analysis, where several options are provided for the analysis of these tables (Fienberg [32], Agresti [33], Johnson and May [34], Poon et al. [35]). From Table 2, one could infer that the different distances handle differently the zero cell. This difference is reflected in the estimate of ${\hat{m}}_{\beta (y_{1}|x)}={\hat{m}}_{\beta_{1}}$, because it is affected by the zero value of $n_{11}$. The strongest control is provided by the Hellinger and symmetric chi-squared distances. All distances estimate the parameters $\pi_{x_{i}}$ similarly, with the bias in their estimation been between 2.7% and 5.2%. The SDs are almost the same for all distances per estimate and their values are ameliorated for N= 10,000.

A referee suggested that in certain cases interest may be centered on smaller samples. We generated 2×3 tables with fixed total sample size of 50 and 70 observations. Table 3 and Table 4 describe the results when the contingency tables were generated under a balanced and an imbalanced design with associated respective Scenarios Ib and IIb. More precisely, Table 3 presents the estimators of the marginal row and column probabilities obtained when PC, HD, SCS and LD distances are used. We notice that the increase in the sample size provides for a decrease in the overall absolute bias in estimation, defined as $\sum_{\ell =1}^{L}\left|{\hat{\theta }}_{\ell }-\theta_{0,\ell }\right|$, where ${\hat{\theta }}_{\ell }$ is the estimate of the *ℓ*-th component of an L×1 vector θ and $\theta_{0,\ell }$ is the corresponding true value. In our case, $\theta^{T}=\left(m_{\beta_{1}},m_{\beta_{2}},\pi_{x_{1}},\pi_{x_{2}},\pi_{x_{3}}\right)$. This observation applies to all distances used in our calculations. Table 4 presents results associated with the imbalanced case. The generated 2×3 tables contain two empty cells ($n_{12}=n_{21}=0$). Once again, for calculating the LD, cells $n_{12}=n_{21}=10^{-8}$. We notice that the bias associated with the estimates is rather large for all the distances, and an increased sample size does not alleviate the observed bias. Basu and Basu [9] have proposed an empty cell penalty for the minimum power-divergence estimators. This penalty leads to estimators with improved small sample properties. See also Alin and Kurt [36] for a discussion of the need of penalization in small samples.

Table 5 provides the results obtained under Scenario III. In this case, the parameter estimates were calculated using the PCS, HD, SCS and LD distances when the 5×5 contingency table was constructed by fixing the row marginal probabilities so that they were all set at 0.20, that is, (0.20,0.20,0.20,0.20,0.20). The column marginals were randomly chosen in the interval [0,1] and summed to 1. In this case, the produced column marginal probabilities were (0.1472,0.2365,0.3196,0.2370,0.0597). The simulation study reveals that the estimates of the parameters $m_{\beta }\left(y|x\right)$’s and $\pi_{x}$’s do not differ substantially from the respective row and column marginal probabilities for any of the four distances utilized. The SDs are approximately the same and they get lower values for larger *N*.

Finally, in Table 6 the data generation was done by exploiting Scenario IV, that is, by having fixed the row marginal probabilities, which were not equal to each other; while, the column marginals were randomly chosen in the interval [0,1] so that they sum to 1. In particular, the row marginal probabilities were fixed at values (0.04,0.20,0.20,0.20,0.36), while the column marginals used were (0.2171,0.1676,0.2347,0.1178,0.2628). When N=100, the value of ${\hat{m}}_{\beta }\left(y_{1}|x\right)={\hat{m}}_{\beta_{1}}$ is not approximately 0.07 and not equal to 0.04 for all distances. However, when N=1000 or N= 10,000, we get better estimates irrespectively of the disparity measure choice. The SDs are approximately the same and they become smaller as the sample size increases.

We also notice from Table 1, Table 5 and Table 6 that in all cases the standard deviation associated with the estimates obtained when we use other than likelihood distances, is approximately the same with the standard deviation that corresponds to the likelihood estimates, thereby showing the asymptotic efficiency of the disparity estimators.

All calculations were performed using the *R* language. Given that the problem described in this section can be viewed as a general non-linear optimization problem, the solnp function of the Rsolnp package (Ye [37]) was used to obtain the aforementioned estimates. For our calculations, we tried using a variety of different initial values (${\hat{\pi }}_{x}^{\left(0\right)}$’s and ${\hat{m}}_{\beta }^{\left(0\right)}\left(y|x\right)$’s); we notice that no matter how the initial values were chosen, the estimates were always pretty similar and very close to the observed values ($n_{i•}/N$ and $n_{•j}/N$ for i,j=1,2,3,4,5). Only the number of iterations needed for convergence is slightly affected. Consequently, random numbers from a Uniform distribution in the interval [0,1] were set as initial values (which were not necessarily summing to 1). The solnp function has a built-in stopping rule and there was no need to set our own stopping rule. We only set the boundary constraints to be in the interval [0,1] for all estimates which were also subject to $\sum \pi_{x}=\sum m_{\beta }\left(y|x\right)=1$.

Other functions may also be used to obtain the estimates. For example, we used the auglag function of the nloptr package with local solvers “lbfgs” or “SLSQP” (Conn et al. [38], Birgin and Martínez [39]) which emulates Augmented Lagrangian multipliers. However, the convergence using the solnp function (the number of iterations was on average 2) was extremely faster than using the auglag function (the average number of iterations was approximately 100). For this reason, the results presented in Table 1, Table 2, Table 3, Table 4, Table 5 and Table 6 were based only on the function solnp.

**Case 2:** *X is discrete and **Y** is continuous*

In this section, we are interested in solving the optimization problem (5) when *X* is discrete, ***Y*** is continuous and X,Y are independent of each other. To evaluate the performance of our procedure, we used Hellinger’s distance, which in this case takes on the following form:

$$HD\left(f^{*},m_{\beta }^{*}\right)=\int \sum_{x}{\left[\sqrt{f_{N}^{*}\left(x,y\right)}-\sqrt{m_{\beta }^{*}\left(x,y\right)}\right]}^{2}dy=\int \sum_{x}{\left[\sqrt{f_{Y}^{*}\left(y\right)\cdot \frac{n_{X}}{N}}-\sqrt{m_{X}\left(x\right)\cdot m_{Y}^{*}\left(y\right)}\right]}^{2}dy.$$

The aim of this simulation is to obtain the minimum Hellinger distance estimators of $\pi_{x}$ and μ assuming (without loss of generality) that $\sigma^{2}$ is known to be equal to 1. All calculations were performed in *R* language.

For this purpose, we generated mixed-type data of size *N* using the package OrdNor (Amatya and Demirtas [40]). More precisely, the data are comprised of one categorical variable *X* with three levels and probability vector (1/3,1/3,1/3), while the continuous part is coming from a trivariate normal distribution; symbolic $Y=\left(Y_{1},Y_{2},Y_{3}\right)∼MVN_{3}\left(\mu ,I_{3}\right)$, where $\mu^{T}=\left(\mu_{1},\mu_{2},\mu_{3}\right)$. We used two different mean vectors: $\mu^{T}=\left(0,0,0\right)$ and $\mu^{T}=\left(0,3,6\right)$. The set of ordinal and normal variables were generated concurrently using an overall correlation matrix Σ, which consists of three components/sub-matrices: $\Sigma_{OO}$, $\Sigma_{ON}$ and $\Sigma_{NN}$, with *O* and *N* corresponding to “Ordinal” and “Normal” variables, respectively. More precisely, the overall correlation matrix Σ used is the following

$$\Sigma =\left(\begin{array}{cccc} 1 & \rho_{ON} & \rho_{ON} & \rho_{ON}\\ \rho_{ON} & 1 & 0 & 0\\ \rho_{ON} & 0 & 1 & 0\\ \rho_{ON} & 0 & 0 & 1 \end{array}\right),$$

where $\Sigma_{OO}=1$, $\Sigma_{NN}=I_{3}$, $\Sigma_{ON}=\left(\begin{array}{ccc} \rho_{ON} & \rho_{ON} & \rho_{ON} \end{array}\right)$ and $\rho_{ON}$ represents the polyserial correlations for the ON combinations (for more information on polyserial correlations refer to Olsson et al. [41]). Since X,Y were assumed to be independent, we set $\rho_{ON}=0.0$. However, we also used weak correlations, say $\rho_{ON}=0.1$ and 0.2, to investigate whether the estimates we receive in these cases remain reasonable.

The kernel function was the multivariate normal density $MVN_{3}\left(0,H\right)$ with H being estimated by the data using the kde function of the ks package (Duong [42]), $m_{Y}^{*}\left(y\right)$ represented the multivariate normal density $MVN_{3}\left(\mu ,\Sigma +H\right)$ and $m_{X}\left(x\right)$ was the multinomial mass function. This choice of smoothing parameter, stemmed from the fact that we were interested in evaluating the performance, in terms of robustness, of standard bandwidth selection.

To solve the optimization problem, the solnp function of the Rsolnp package (Ye [37]) was used. Specifically, the initial values set for the probabilities $\pi_{x_{1}},\pi_{x_{2}},\pi_{x_{3}}$ associated with the *X* variable were random uniform numbers in the interval [0,1], while the initial values for the means $\mu_{y_{1}},\mu_{y_{2}},\mu_{y_{3}}$ were random numbers in the interval $\left[Q1\left(Y_{i}\right),Q3\left(Y_{i}\right)\right]$ for i=1,2,3, where Q1 and Q3 stand for the respective 25th and the 75th quantile per component of the continuous part. Following the same procedure with the one of Basu and Lindsay [2] in the univariate continuous case, here (in the mixed-case) the numerical evaluation of the integrals was also done on the basis of the Simpson’s 1/3rd rule using the sintegral function of the Bolstad2 package (Bolstad [43]). Moreover, we calculated the mean values, the SDs, as well as the percentages of bias of the mean and the probability vectors for three different sample sizes: N=100; N=1000 and N=1500 over 1000 MC replications. The bias is defined as the difference of the estimates from their “true” values, that is, $bias\left(\mu_{y_{i}}\right)={\hat{\mu }}_{y_{i}}-\mu_{i}$ and $bias\left(\pi_{x_{i}}\right)={\hat{\pi }}_{x_{i}}-1/3$ for i=1,2,3. The results are shown in Table 7 and Table 8.

In particular, Table 7 illustrates the mean values, the SDs and the bias percentages of the corresponding minimum Hellinger distance estimators, over 1000 MC replications, for the three different sample sizes and polyserial correlations, when $\mu ={\left(0,0,0\right)}^{T}$. The estimates for the $\pi_{x_{i}}$ are approximately equal to 1/3=0.333, while the $\mu_{y_{i}}$ estimates are almost zero, even in the cases of weak correlations. When $\rho_{ON}=0.0$, the sample size choice does not seem to affect the values of the estimates either overall or per component of X,Y variables. Specifically, we observe that the total absolute bias, computed as the sum of the individual component-wise absolute biases of the vectors $\pi^{T}=\left(\pi_{1},\pi_{2},\pi_{3}\right)$ and $\mu^{T}=\left(\mu_{1},\mu_{2},\mu_{3}\right)$ are approximately the same, with larger samples providing slightly less biases at the expense of a higher computational cost.

In Table 8, analogous results are presented with the difference that the mean vector used was $\mu ={\left(0,3,6\right)}^{T}$. The $\pi_{x_{i}}$ estimates are very close to 1/3(=0.333) for all *X* components, no matter which sample size or correlation is used. On the contrary, the interpretation of the $\mu_{i}$ estimates slightly differs in this case. We also calculated the overall absolute bias as well as the individual, per parameter, absolute biases. In this case, larger samples clearly provide estimates with smaller bias for both parameter vectors π, μ and for both cases, the case of independence as well as the case of weak correlations. However, the computational time increases.

In what follows, we also present -for illustration purposes- a small simulation example using a mixed-type, contaminated data set of size N=1000, which was generated using OrdNor package setting $\rho_{ON}=0.0$. Once again, the data were comprised of one categorical variable *X* with three levels and probability vector (1/3,1/3,1/3), and a trivariate continuous vector $Y=(Y_{1},Y_{2},Y_{3})$. The contamination is happening only in the continuous part on the basis of α∈{1.00,0.95,0.90,0.85,0.80}, as follows: $Y∼\alpha \times MVN_{3}\left(0,I_{3}\right)+\left(1-\alpha \right)\times MVN_{3}\left(\mu ,I_{3}\right)$, where $\mu^{T}=\left(3,3,3\right)$. This means that, $N_{1}=\alpha \times N$ data were generated with *Y* coming from multivaraiate standard normal and the remaining $N_{2}=N-N_{1}$ subset of the data followed a multivaraiate normal distribution with mean vector $\mu^{T}=\left(3,3,3\right)$. It goes without saying that when α=1.00, there is no contamination. Here, we are still considering the same optimization problem with the one described above and, consequently, we are interested in evaluating the minimum Hellinger distance estimators over 1000 MC replications by examining/studying to what extend the contamination level affects these estimates.

As indicated from Table 9, when there is no contamination in the data (α=1.00), the estimates for the $\pi_{x_{i}}$s are almost equal to 1/3, while the $\mu_{y}$’s estimates are almost equal to zero. As the data become more contaminated (i.e., the value of α decreases), the minimum disparity estimators corresponding to *X* variable remain pretty consistent with their true values. However, this is not the case with the estimates for the $\mu_{y_{i}}$s, which deteriorate as the value of the contamination level α shifts from the target/null value, that is 1.00.

The mean parameters are estimated with reasonable bias (maximum bias is 9% for the second component of the mean) when α=0.95, that is the contamination is 5%. When the contamination is 10%, the bias of the mean components is relatively high but still below 19%. With higher contamination, the percentage of bias in the mean components is in the interval [28.3%,47%]. This is the result of using standard density estimation to obtain the smoothing parameters for the different mean components. Smaller values of these component smoothing parameters result in substantial bias reduction.

We also looked at the case where the continuous model was contaminated by a trivariate normal with mean $\mu^{T}=\left(1.5,1.5,1.5\right)$ and covariance matrix I. In this case (results not shown), when the contamination is 5% the maximum bias of the mean components is 6.6%, while when the contamination is 10% the maximum bias of the mean components is 13.5%. Again, in this case the bandwidth parameters were obtained by fitting a unimodal density to the data.

The above results are not surprising. A judicious selection of the smoothing parameter decreases the bias of the component estimates of the mean. Agostinelli and Markatou [44] provide suggestions of how to select the smoothing parameter that can be extended and applied in this context.

## 8. Discussion and Conclusions

In this paper, we discuss Pearson residual systems that conform to the measurement scale of the data. We place emphasis on the mixed-scale measurements scenario, which is equivalent to having both discrete (categorical or nominal) and continuous type random variables, and obtain robust estimators of the parameters of the joint probability distribution that describes those variables. We show that, disparity methods can be used to actually control against model misspecification and the presence of outliers, and these methods provide reasonable results.

The scale and nature of measurement of the data imposes additional challenges, both computationally and statistically. Detecting outliers in this multidimensional space is an open research question (Eiras-Franco et al. [45]). The concept of outliers has a long history in the field of statistics and outlier detection methods have broad applications in many scientific fields such as security (Diehl and Hampshire [46], Portnoy et al. [47]), health care (Tran et al. [48]) and insurance (Konijn and Kowalczyk [49]) to mention just a few.

Classical outlier detection methods are largely designed for single measurement scale data. Handling mixed measurement scale is a challenge with few works coming from both, the field of statistics (Fraley and Wilkinson [50], Wilkinson [51]) and the fields of engineering and computer science (Do et al. [52], Koufakou et al. [53]). All these works use some version of a probabilistic outlier, either looking for regions in the space of data that have low density (Do et al. [52], Koufakou et al. [53]) or by attaching a probability, under a model, to the suspicious data point (Fraley and Wilkinson [50], Wilkinson [51]).

Our concept of a probabilistic outlier discussed here and expressed via the construction of appropriate Pearson residuals can unify the different measurement scales, and the class of disparity functions discussed above can provide estimators for the model parameters that are not influenced unduly by potential outliers.

One of the important parameters that controls the robustness of these methods is the smoothing parameter(s) used to compute the density estimator of the continuous part of the model. In our computations, we use standard smoothing parameters obtained from utilizing appropriate *R* functions for density estimation. The results show that, depending on the level of contamination and the type of contaminating probability model, the performance of the methods is satisfactory. Specifically, a small simulation study using the model reported in the caption of Table 9 shows that the overall bias associated with the mean components of the standard multivariate normal model is low when contamination with a multivariate normal model with mean components equal to 3 is less than or equal to 10%. But even in this case, when the percentage of contamination is greater than 10%, the bias increases when the smoothing parameter used is the one obtained from the *R* density function. Here, smaller values of the smoothing parameter guarantee reduction of the bias.

Devising rules for selecting the smoothing parameter(s) in the context of mixed-scale measurements that can guarantee robustness for larger than 5% levels of contamination may be possible. However, it is the opinion of the authors that greater levels of data inhomogeneity may indicate model failure, a case where assessing model goodness of fit is of importance.

## Figures and Tables

**Table 1:** Scenario Ia: Means and standard deviations (SDs) of 4 distances (PCS,HD,SCS,LD). A 5×5 contingency table was generated having fixed the total sample size *N* under a balanced design with $n_{ij}\neq 0,\;\forall \;i,j=1,2,3,4,5$. The number of Monte Carlo (MC) replications used is 10,000.

*N*	Statistical Distance	Summary	Estimates									
			Means and SDs over 10,000 Replications									
			${\hat{m}}_{\beta_{1}}$	${\hat{m}}_{\beta_{2}}$	${\hat{m}}_{\beta_{3}}$	${\hat{m}}_{\beta_{4}}$	${\hat{m}}_{\beta_{5}}$	${\hat{\pi }}_{x_{1}}$	${\hat{\pi }}_{x_{2}}$	${\hat{\pi }}_{x_{3}}$	${\hat{\pi }}_{x_{4}}$	${\hat{\pi }}_{x_{5}}$
100	PCS	Mean	0.199	0.199	0.201	0.201	0.200	0.201	0.200	0.199	0.200	0.201
		SD	0.038	0.041	0.039	0.039	0.039	0.038	0.038	0.037	0.038	0.038
	HD	Mean	0.199	0.200	0.200	0.200	0.201	0.200	0.200	0.200	0.200	0.200
		SD	0.037	0.041	0.037	0.037	0.037	0.037	0.037	0.035	0.036	0.037
	SCS	Mean	0.199	0.201	0.200	0.200	0.200	0.200	0.200	0.199	0.200	0.201
		SD	0.037	0.041	0.038	0.038	0.038	0.032	0.033	0.030	0.031	0.032
	LD	Mean	0.199	0.200	0.200	0.200	0.200	0.200	0.002	0.200	0.200	0.200
		SD	0.035	0.039	0.036	0.036	0.036	0.035	0.036	0.036	0.034	0.035
1000	PCS	Mean	0.200	0.200	0.200	0.200	0.200	0.200	0.200	0.200	0.200	0.200
		SD	0.014	0.015	0.016	0.016	0.014	0.017	0.015	0.015	0.013	0.016
	HD	Mean	0.200	0.200	0.200	0.200	0.200	0.200	0.200	0.200	0.200	0.200
		SD	0.013	0.015	0.013	0.013	0.013	0.013	0.012	0.012	0.012	0.013
	SCS	Mean	0.200	0.200	0.200	0.200	0.200	0.200	0.200	0.200	0.200	0.200
		SD	0.014	0.015	0.013	0.013	0.013	0.008	0.009	0.011	0.012	0.008
	LD	Mean	0.200	0.200	0.200	0.200	0.200	0.200	0.200	0.200	0.200	0.200
		SD	0.013	0.015	0.013	0.013	0.013	0.013	0.013	0.012	0.012	0.013
10,000	PCS	Mean	0.200	0.200	0.200	0.200	0.200	0.200	0.200	0.200	0.200	0.200
		SD	0.008	0.007	0.006	0.006	0.009	0.010	0.010	0.007	0.008	0.006
	HD	Mean	0.200	0.200	0.200	0.200	0.200	0.200	0.200	0.200	0.200	0.200
		SD	0.004	0.005	0.004	0.004	0.004	0.004	0.004	0.004	0.004	0.004
	SCS	Mean	0.200	0.200	0.200	0.200	0.200	0.200	0.200	0.200	0.200	0.200
		SD	0.004	0.005	0.004	0.004	0.004	0.007	0.005	0.008	0.008	0.004
	LD	Mean	0.200	0.200	0.200	0.200	0.200	0.200	0.200	0.200	0.200	0.200
		SD	0.004	0.005	0.004	0.004	0.004	0.004	0.004	0.004	0.004	0.004

**Table 2:** Scenario IIa Means and SDs of 4 distances (PCS,HD,SCS,LD). A 5×5 contingency table was generated having fixed the total sample size *N* under an imbalanced design with $n_{11}=0$. The number of MC replications used is 10,000.

*N*	Statistical Distance	Summary	Estimates									
			Means and SDs over 10,000 Replications									
			${\hat{m}}_{\beta_{1}}$	${\hat{m}}_{\beta_{2}}$	${\hat{m}}_{\beta_{3}}$	${\hat{m}}_{\beta_{4}}$	${\hat{m}}_{\beta_{5}}$	${\hat{\pi }}_{x_{1}}$	${\hat{\pi }}_{x_{2}}$	${\hat{\pi }}_{x_{3}}$	${\hat{\pi }}_{x_{4}}$	${\hat{\pi }}_{x_{5}}$
100	PCS	Mean	0.052	0.197	0.198	0.198	0.355	0.165	0.173	0.172	0.245	0.245
		SD	0.028	0.045	0.044	0.044	0.053	0.041	0.039	0.044	0.044	0.047
	HD	Mean	0.026	0.202	0.202	0.202	0.368	0.156	0.168	0.168	0.254	0.254
		SD	0.019	0.049	0.045	0.045	0.054	0.041	0.042	0.041	0.046	0.049
	SCS	Mean	0.033	0.209	0.209	0.209	0.340	0.166	0.172	0.171	0.245	0.246
		SD	0.022	0.047	0.045	0.045	0.051	0.036	0.036	0.033	0.038	0.040
	LD	Mean	0.040	0.200	0.200	0.200	0.360	0.160	0.170	0.170	0.250	0.250
		SD	0.020	0.043	0.040	0.040	0.048	0.037	0.038	0.036	0.042	0.044
1000	PCS	Mean	0.044	0.197	0.197	0.197	0.365	0.164	0.170	0.170	0.248	0.248
		SD	0.011	0.017	0.014	0.014	0.018	0.013	0.014	0.013	0.015	0.015
	HD	Mean	0.034	0.203	0.202	0.202	0.359	0.156	0.170	0.170	0.252	0.252
		SD	0.005	0.015	0.013	0.013	0.016	0.011	0.012	0.012	0.013	0.014
	SCS	Mean	0.038	0.210	0.210	0.210	0.332	0.166	0.169	0.169	0.248	0.248
		SD	0.006	0.015	0.014	0.014	0.016	0.014	0.013	0.011	0.013	0.014
	LD	Mean	0.040	0.200	0.200	0.200	0.360	0.160	0.170	0.170	0.250	0.250
		SD	0.006	0.015	0.013	0.013	0.016	0.012	0.012	0.011	0.013	0.014
10,000	PCS	Mean	0.044	0.197	0.196	0.196	0.367	0.164	0.170	0.170	0.248	0.248
		SD	0.002	0.006	0.007	0.007	0.010	0.007	0.006	0.005	0.007	0.008
	HD	Mean	0.034	0.203	0.202	0.202	0.359	0.156	0.171	0.171	0.252	0.252
		SD	0.002	0.005	0.004	0.004	0.005	0.004	0.004	0.004	0.004	0.005
	SCS	Mean	0.038	0.210	0.210	0.210	0.332	0.166	0.169	0.169	0.248	0.248
		SD	0.002	0.005	0.004	0.004	0.005	0.007	0.006	0.004	0.006	0.006
	LD	Mean	0.040	0.200	0.200	0.200	0.360	0.160	0.170	0.170	0.250	0.250
		SD	0.002	0.005	0.004	0.004	0.005	0.004	0.004	0.004	0.004	0.004

**Table 3:** Scenario Ib: Means and Biases of 4 distances (PCS,HD,SCS,LD). A 2×3 contingency table was generated having fixed the total sample size *N* under a balanced design with $n_{ij}\neq 0,\;\forall \;i=1,2,\;j=1,2,3$. The number of MC replications used is 10,000.

*N*	Statistical Distance	Summary	Estimates				
			Means and Biases over 10,000 Replications				
			${\hat{m}}_{\beta_{1}}$	${\hat{m}}_{\beta_{2}}$	${\hat{\pi }}_{x_{1}}$	${\hat{\pi }}_{x_{2}}$	${\hat{\pi }}_{x_{3}}$
50	PCS	Mean	0.5008	0.4992	0.3339	0.3336	0.3325
		Abs.Biases	0.0008	0.0008	0.0006	0.0003	0.0009
		Overall Bias			0.0034		
	HD	Mean	0.5008	0.4992	0.3339	0.3335	0.3326
		Abs.Biases	0.0008	0.0008	0.0006	0.0002	0.0007
		Overall Bias			0.0031		
	SCS	Mean	0.5007	0.4993	0.3338	0.3335	0.3326
		Abs.Biases	0.0007	0.0007	0.0005	0.0002	0.0007
		Overall Bias			0.0028		
	LD	Mean	0.5008	0.4992	0.3339	0.3335	0.3326
		Abs.Biases	0.0008	0.0008	0.0006	0.0002	0.0008
		Overall Bias			0.0032		
70	PCS	Mean	0.4998	0.5002	0.3333	0.3331	0.3337
		Abs.Biases	0.0002	0.0002	0.0001	0.0003	0.0003
		Overall Bias			0.0011		
	HD	Mean	0.4998	0.5002	0.3333	0.3330	0.3336
		Abs.Biases	0.0002	0.0002	0.0000	0.0003	0.0003
		Overall Bias			0.0009		
	SCS	Mean	0.4998	0.5002	0.3334	0.3331	0.3335
		Abs.Biases	0.0002	0.0002	0.0000	0.0002	0.0002
		Overall Bias			0.0008		
	LD	Mean	0.4999	0.5001	0.3333	0.3330	0.3336
		Abs.Biases	0.0001	0.0001	0.0000	0.0003	0.0003
		Overall Bias			0.0009		

**Table 4:** Scenario IIb: Means and Biases of 4 distances (PCS,HD,SCS,LD). A 2×3 contingency table was generated having fixed the total sample size *N* under an imbalanced design with $n_{12}=n_{21}=0$. The number of MC replications used is 10,000.

*N*	Statistical Distance	Summary	Estimates				
			Means and Biases over 10,000 Replications				
			${\hat{m}}_{\beta_{1}}$	${\hat{m}}_{\beta_{2}}$	${\hat{\pi }}_{x_{1}}$	${\hat{\pi }}_{x_{2}}$	${\hat{\pi }}_{x_{3}}$
50	PCS	Mean	0.6391	0.3609	0.3489	0.2278	0.4234
		Abs.Biases	0.0276	0.0276	0.0155	0.0611	0.0766
		Overall Bias			0.2084		
	HD	Mean	0.7815	0.2185	0.3346	0.0497	0.6157
		Abs.Biases	0.1149	0.1149	0.0013	0.1170	0.1157
		Overall Bias			0.4638		
	SCS	Mean	0.6420	0.3580	0.3510	0.2726	0.3765
		Abs.Biases	0.0247	0.0247	0.0176	0.1059	0.1235
		Overall Bias			0.2964		
	LD	Mean	0.6677	0.3323	0.3342	0.1660	0.4998
		Abs.Biases	0.0010	0.0010	0.0009	0.0007	0.0002
		Overall Bias			0.0038		
70	PCS	Mean	0.6377	0.3623	0.3483	0.2297	0.4220
		Abs.Biases	0.0290	0.0290	0.0150	0.0631	0.0780
		Overall Bias			0.2141		
	HD	Mean	0.7812	0.2188	0.3328	0.0491	0.6180
		Abs.Biases	0.1145	0.1145	0.0005	0.1175	0.1180
		Overall Bias			0.4650		
	SCS	Mean	0.6395	0.3605	0.3505	0.2739	0.3756
		Abs.Biases	0.0271	0.0271	0.0172	0.1072	0.1244
		Overall Bias			0.3030		
	LD	Mean	0.6657	0.3343	0.3331	0.1671	0.4998
		Abs.Biases	0.0010	0.0010	0.0002	0.0004	0.0002
		Overall Bias			0.0028		

**Table 5:** Scenario III: Means and SDs of 4 distances (PCS,HD,SCS,LD). A 5×5 contingency table was generated having fixed the row marginal probabilities at (0.20, 0.20, 0.20, 0.20, 0.20). The number of MC replications used is 10,000.

*N*	Statistical Distance	Summary	Estimates									
			Means and SDs over 10,000 Replications									
			${\hat{m}}_{\beta_{1}}$	${\hat{m}}_{\beta_{2}}$	${\hat{m}}_{\beta_{3}}$	${\hat{m}}_{\beta_{4}}$	${\hat{m}}_{\beta_{5}}$	${\hat{\pi }}_{x_{1}}$	${\hat{\pi }}_{x_{2}}$	${\hat{\pi }}_{x_{3}}$	${\hat{\pi }}_{x_{4}}$	${\hat{\pi }}_{x_{5}}$
100	PCS	Mean	0.199	0.200	0.200	0.200	0.201	0.153	0.230	0.302	0.229	0.086
		SD	0.037	0.037	0.037	0.037	0.037	0.034	0.039	0.043	0.039	0.023
	HD	Mean	0.200	0.200	0.200	0.200	0.200	0.147	0.230	0.311	0.230	0.082
		SD	0.039	0.040	0.039	0.039	0.040	0.033	0.043	0.037	0.042	0.019
	SCS	Mean	0.200	0.200	0.200	0.200	0.200	0.153	0.230	0.302	0.230	0.085
		SD	0.039	0.085	0.038	0.038	0.038	0.033	0.039	0.043	0.039	0.022
	LD	Mean	0.200	0.200	0.200	0.200	0.200	0.150	0.230	0.307	0.230	0.083
		SD	0.038	0.038	0.038	0.038	0.038	0.033	0.041	0.045	0.040	0.019
1000	PCS	Mean	0.200	0.200	0.200	0.200	0.200	0.148	0.236	0.319	0.236	0.061
		SD	0.013	0.013	0.013	0.013	0.014	0.012	0.014	0.017	0.015	0.011
	HD	Mean	0.200	0.200	0.200	0.200	0.200	0.147	0.237	0.320	0.237	0.059
		SD	0.013	0.013	0.013	0.013	0.013	0.011	0.014	0.015	0.014	0.008
	SCS	Mean	0.200	0.200	0.200	0.200	0.200	0.148	0.236	0.319	0.237	0.060
		SD	0.015	0.015	0.015	0.015	0.015	0.011	0.014	0.016	0.014	0.013
	LD	Mean	0.200	0.200	0.200	0.200	0.200	0.147	0.237	0.320	0.237	0.059
		SD	0.013	0.013	0.013	0.013	0.013	0.011	0.014	0.015	0.013	0.008
10,000	PCS	Mean	0.200	0.200	0.200	0.200	0.200	0.147	0.236	0.320	0.237	0.060
		SD	0.006	0.006	0.006	0.006	0.006	0.008	0.006	0.011	0.006	0.008
	HD	Mean	0.200	0.200	0.200	0.200	0.200	0.147	0.236	0.320	0.237	0.060
		SD	0.004	0.004	0.004	0.004	0.004	0.004	0.004	0.005	0.004	0.002
	SCS	Mean	0.200	0.200	0.200	0.200	0.200	0.147	0.236	0.320	0.237	0.060
		SD	0.005	0.005	0.005	0.005	0.005	0.004	0.006	0.008	0.006	0.008
	LD	Mean	0.200	0.200	0.200	0.200	0.200	0.147	0.236	0.320	0.237	0.060
		SD	0.004	0.004	0.004	0.004	0.004	0.004	0.005	0.005	0.005	0.002

**Table 6:** Scenario IV: Means and SDs of 4 distances (PCS,HD,SCS,LD). A 5×5 contingency table was generated having fixed the row marginal probabilities at (0.04, 0.20, 0.20, 0.20, 0.36). The number of MC replications used is 10,000.

*N*	Statistical Distance	Summary	Estimates									
			Means and SDs over 10,000 Replications									
			${\hat{m}}_{\beta_{1}}$	${\hat{m}}_{\beta_{2}}$	${\hat{m}}_{\beta_{3}}$	${\hat{m}}_{\beta_{4}}$	${\hat{m}}_{\beta_{5}}$	${\hat{\pi }}_{x_{1}}$	${\hat{\pi }}_{x_{2}}$	${\hat{\pi }}_{x_{3}}$	${\hat{\pi }}_{x_{4}}$	${\hat{\pi }}_{x_{5}}$
100	PCS	Mean	0.074	0.197	0.197	0.197	0.335	0.214	0.173	0.228	0.132	0.253
		SD	0.022	0.037	0.038	0.038	0.045	0.038	0.035	0.039	0.031	0.041
	HD	Mean	0.070	0.194	0.195	0.195	0.346	0.215	0.170	0.231	0.126	0.258
		SD	0.015	0.039	0.039	0.039	0.048	0.041	0.037	0.042	0.030	0.044
	SCS	Mean	0.074	0.194	0.195	0.195	0.342	0.214	0.173	0.229	0.131	0.253
		SD	0.015	0.039	0.039	0.039	0.048	0.038	0.035	0.040	0.030	0.041
	LD	Mean	0.071	0.195	0.196	0.196	0.342	0.214	0.172	0.230	0.128	0.256
		SD	0.015	0.037	0.038	0.038	0.046	0.040	0.036	0.041	0.030	0.042
1000	PCS	Mean	0.042	0.200	0.200	0.200	0.358	0.217	0.168	0.234	0.119	0.262
		SD	0.011	0.014	0.013	0.013	0.017	0.014	0.013	0.014	0.014	0.015
	HD	Mean	0.039	0.200	0.200	0.200	0.361	0.217	0.167	0.235	0.118	0.263
		SD	0.006	0.013	0.013	0.013	0.015	0.013	0.012	0.013	0.010	0.014
	SCS	Mean	0.039	0.200	0.200	0.200	0.361	0.217	0.168	0.234	0.118	0.263
		SD	0.007	0.013	0.013	0.013	0.016	0.016	0.013	0.014	0.010	0.015
	LD	Mean	0.040	0.200	0.200	0.200	0.360	0.217	0.167	0.235	0.118	0.263
		SD	0.006	0.013	0.013	0.013	0.015	0.013	0.012	0.013	0.010	0.014
10,000	PCS	Mean	0.040	0.200	0.200	0.200	0.360	0.217	0.167	0.235	0.118	0.263
		SD	0.008	0.005	0.007	0.007	0.009	0.006	0.005	0.005	0.007	0.006
	HD	Mean	0.040	0.200	0.200	0.200	0.360	0.217	0.167	0.235	0.118	0.263
		SD	0.002	0.004	0.004	0.004	0.005	0.004	0.004	0.004	0.003	0.004
	SCS	Mean	0.040	0.200	0.200	0.200	0.360	0.217	0.167	0.235	0.118	0.263
		SD	0.002	0.004	0.004	0.004	0.005	0.006	0.005	0.007	0.003	0.008
	LD	Mean	0.040	0.200	0.200	0.200	0.360	0.217	0.167	0.235	0.118	0.263
		SD	0.002	0.004	0.004	0.004	0.005	0.004	0.004	0.005	0.003	0.005

**Table 7:** Means, Absolute Biases and Overall Absolute Bias of the Hellinger’s distance (HD). The data were concurrently generated with a given correlation structure (an overall correlation matrix Σ) and consist of a discrete variable *X* with marginal probability vector (1/3,1/3,1/3) and a continuous vector $Y=\left(Y_{1},Y_{2},Y_{3}\right)∼MVN_{3}\left(\mu ,I_{3}\right)$, where $\mu^{T}=\left(0,0,0\right)$ and $I_{3}$ is a (3×3) identity matrix. The number of MC replications used is 1000.

$\rho_{\mathrm{ON}}$	*N*	Summary	Estimates					
			Means, Biases over 1000 Replications					
			${\hat{\pi }}_{x_{1}}$	${\hat{\pi }}_{x_{2}}$	${\hat{\pi }}_{x_{3}}$	${\hat{\mu }}_{y_{1}}$	${\hat{\mu }}_{y_{2}}$	${\hat{\mu }}_{y_{3}}$
0.0	50	Mean	0.332	0.340	0.329	0.016	0.011	−0.011
		Abs. Biases	0.001	0.007	0.004	0.016	0.011	0.011
		Overall Bias	0.050					
	100	Mean	0.330	0.350	0.320	0.017	−0.018	−0.010
		Abs. Biases	0.003	0.017	0.013	0.017	0.018	0.010
		Overall Bias	0.078					
	1000	Mean	0.324	0.337	0.339	0.001	−0.008	0.007
		Abs. Biases	0.009	0.004	0.006	0.001	0.008	0.007
		Overall Bias	0.035					
0.1	50	Mean	0.351	0.320	0.329	−0.006	0.003	0.005
		Abs. Biases	0.018	0.013	0.004	0.006	0.003	0.005
		Overall Bias	0.049					
	100	Mean	0.330	0.323	0.347	0.001	0.005	−0.004
		Abs. Biases	0.003	0.010	0.014	0.001	0.005	0.004
		Overall Bias	0.037					
	1000	Mean	0.327	0.343	0.330	−0.021	0.008	0.003
		Abs. Biases	0.006	0.010	0.003	0.021	0.008	0.003
		Overall Bias	0.051					

**Table 8:** Means, Absolute Biases and Overall Absolute Bias of the Hellinger’s distance (HD). The data were concurrently generated with a given correlation structure (an overall correlation matrix Σ) and consist of a discrete variable *X* with marginal probability vector (1/3,1/3,1/3) and a continuous vector $Y=\left(Y_{1},Y_{2},Y_{3}\right)∼MVN_{3}\left(\mu ,I_{3}\right)$, where $\mu^{T}=\left(0,3,6\right)$ and $I_{3}$ is a (3×3) identity matrix. The number of MC replications used is 1000.

$\rho_{\mathrm{ON}}$	*N*	Summary	Estimates					
			Means, Biases over 1000 Replications					
			${\hat{\pi }}_{x_{1}}$	${\hat{\pi }}_{x_{2}}$	${\hat{\pi }}_{x_{3}}$	${\hat{\mu }}_{y_{1}}$	${\hat{\mu }}_{y_{2}}$	${\hat{\mu }}_{y_{3}}$
0.0	50	Mean	0.340	0.328	0.332	−0.004	2.606	5.227
		Abs. Biases	0.007	0.005	0.001	0.004	0.394	0.773
		Overall Bias	1.184					
	100	Mean	0.313	0.350	0.337	−0.004	2.777	5.593
		Abs. Biases	0.020	0.017	0.004	0.004	0.223	0.407
		Overall Bias	0.675					
	1000	Mean	0.338	0.334	0.328	0.012	2.972	5.958
		Abs. Biases	0.005	0.001	0.005	0.012	0.028	0.042
		Overall Bias	0.093					
0.1	50	Mean	0.347	0.323	0.330	−0.021	2.628	5.249
		Abs. Biases	0.014	0.010	0.003	0.021	0.372	0.751
		Overall Bias	1.171					
	100	Mean	0.317	0.343	0.340	0.017	2.817	5.615
		Abs. Biases	0.016	0.010	0.007	0.017	0.183	0.385
		Overall Bias	0.618					
	1000	Mean	0.334	0.320	0.346	−0.013	2.988	5.956
		Abs. Biases	0.001	0.013	0.013	0.013	0.012	0.044
		Overall Bias	0.096					
0.2	50	Mean	0.324	0.333	0.343	−0.004	2.589	5.240
		Abs. Biases	0.009	0.000	0.010	0.004	0.411	0.760
		Overall Bias	1.194					
	100	Mean	0.329	0.350	0.321	0.024	2.763	5.549
		Abs. Biases	0.004	0.017	0.012	0.024	0.237	0.451
		Overall Bias	0.745					
	1000	Mean	0.337	0.344	0.319	−0.011	2.971	5.951
		Abs. Biases	0.004	0.011	0.014	0.019	0.029	0.049
		Overall Bias	0.118					

**Table 9:** Means and SDs of the Hellinger’s distance (HD). The data were concurrently generated with a given correlation structure (an overall correlation matrix Σ) and consist of a discrete variable *X* with marginal probability vector (1/3,1/3,1/3) and a continuous trivariate vector $Y=\left(Y_{1},Y_{2},Y_{3}\right)∼\alpha \times MVN_{3}\left(0,I_{3}\right)+\left(1-\alpha \right)\times MVN_{3}\left(\mu ,I_{3}\right)$, where $\mu^{T}=\left(3,3,3\right)$, $I_{3}$ is a (3×3) identity matrix and α=1.00(0.05)0.80 indicates the contamination level. The number of MC replications used is 1000.

$\rho_{\mathrm{ON}}$	*N*	α	Summary	Estimates					
				Means and SDs over 1000 Replications					
				${\hat{\pi }}_{x_{1}}$	${\hat{\pi }}_{x_{2}}$	${\hat{\pi }}_{x_{3}}$	${\hat{\mu }}_{y_{1}}$	${\hat{\mu }}_{y_{2}}$	${\hat{\mu }}_{y_{3}}$
0.0	1000	1.00	Mean	0.324	0.337	0.339	0.001	−0.008	0.007
			SD	0.293	0.293	0.298	0.378	0.378	0.386
		0.95	Mean	0.327	0.326	0.347	0.068	0.090	0.079
			SD	0.304	0.299	0.309	0.413	0.413	0.413
		0.90	Mean	0.318	0.331	0.351	0.188	0.170	0.189
			SD	0.300	0.305	0.306	0.443	0.450	0.436
		0.85	Mean	0.324	0.337	0.339	0.292	0.283	0.312
			SD	0.293	0.293	0.297	0.484	0.487	0.491
		0.80	Mean	0.324	0.337	0.338	0.447	0.436	0.470
			SD	0.293	0.293	0.297	0.552	0.547	0.559

## Abbreviations

The following abbreviations are used in this manuscript:

ALT	Alanine Aminotransferase
HD	Twice-Squared Hellinger’s Disparity
LD	Likelihood Disparity
MC	Monte Carlo Replications
MDE	Minimum Distance Estimators
MLE	Maximum Likelihood Estimator
PCS	Pearson’s Chi-Squared Disparity Divided by 2
PWD	Power Divergence Disparity
RAF	Residual Adjustment Function
SCS	Symmetric Chi-Squared Disparity
SD	Standard Deviation

## Appendix A

### Appendix A.1. Proof of Proposition 3

**Proof.** The equations (4) are obtained from solving optimization problem (3). To solve this problem we need to form the corresponding Langrangian, which is

$$\sum_{x,y}G\left(\delta \left(x,y\right)\right)m_{\beta }\left(y|x\right)\pi_{x}-\lambda \left(\sum \pi_{x}-1\right).$$

(i) Let $\nabla_{\beta }$ denote gradient with respect to β. The estimators of β are obtained as solutions of the set of equations:

$$\nabla_{\beta }\left\{\sum_{x,y}G\left(\delta \left(x,y\right)\right)m_{\beta }\left(y|x\right)\pi_{x}-\lambda \left(\sum \pi_{x}-1\right)\right\}=0,$$

which can be equivalently expressed as follows,

$$\sum_{x,y}\pi_{x}\left[\nabla_{\beta }G\left(\delta \left(x,y\right)\right)\right]m_{\beta }\left(y|x\right)+\sum_{x,y}\pi_{x}G\left(\delta \left(x,y\right)\right)\nabla_{\beta }\left(y|x\right)=0.$$

Notice that the $\nabla_{\beta }$ of G(δ(x,y)) is given by

$$\nabla_{\beta }G\left(\delta \left(x,y\right)\right)=-G^{\prime }\left(\delta \left(x,y\right)\right)\left(\delta \left(x,y\right)+1\right)\;u\left(y|x;\beta \right),$$

where the superscript "’" denote derivative with respect to δ, δ(x,y) is the Pearson residual and

$$u\left(y|x;\beta \right)=\frac{\nabla_{\beta }m_{\beta }\left(y|x\right)}{m_{\beta }\left(y|x\right)}=\nabla_{\beta }\ln\left[m_{\beta }\left(y|x\right)\right]$$

is the score for β in the conditional distribution of y given x. Therefore,

$$\sum_{x,y}A\left(\delta \left(x,y\right)\right)\pi_{x}u\left(y|x;\beta \right)m_{\beta }\left(y|x\right)=0,$$

where

$$A\left(\delta \left(x,y\right)\right)=G^{\prime }\left(\delta \left(x,y\right)\right)\left[\delta \left(x,y\right)+1\right]-G\left(\delta \left(x,y\right)\right).$$

By making use of the fact that $\sum_{x}\pi_{x}\nabla_{\beta }m_{\beta }\left(y|x\right)=0$, the resulting equations can represented as

$$\sum_{x,y}\frac{A(\delta (x,y))+1}{\delta (x,y)+1}n_{x,y}u\left(y|x;\beta \right)=0,$$

or equivalently,

$$\sum_{x,y}w\left(\delta \left(x,y\right)\right)n_{x,y}u\left(y|x;\beta \right)=0.$$

Without loss of generality, we can take,

$$w\left(\delta \left(x,y\right)\right)=\min\left\{\frac{{\left[A\left(\delta \left(x,y\right)\right)+1\right]}^{+}}{\delta (x,y)+1},1\right\},w\left(\delta \left(x,y\right)\right)\leq 1.$$

(ii) We now need to obtain ${\hat{\pi }}_{x}$, which can be obtained by setting the gradient of formula with respect to $\pi_{z}$ equal to zero, that is, by the following equations:

$$\sum_{y}G^{\prime }\left(\delta \left(z,y\right)\right)\left[\nabla \pi_{z}\delta \left(z,y\right)\right]m_{\beta }\left(y|z\right)\pi_{z}+\sum_{y}G\left(\delta \left(z,y\right)\right)m_{\beta }\left(y|z\right)-\lambda =0.$$

Recording $A\left(\delta \left(z,y\right)\right)=G^{\prime }\left(\delta \left(z,y\right)\right)\left[\delta \left(z,y\right)+1\right]-G\left(\delta \left(z,y\right)\right)$ and $\delta \left(z,y\right)+1=\frac{n_{z,y}/n}{m_{\beta }\left(y|z\right)\pi_{z}}$, the above equations are reduced to,

$$\sum_{y}A\left(\delta \left(z,y\right)\right)m_{\beta }\left(z,y\right)\frac{1}{\pi_{z}}+\lambda =0$$

and we readily conclude that,

$$\pi_{z}=-\frac{1}{\lambda }\sum_{y}A\left(\delta \left(z,y\right)\right)m\left(z,y\right),\forall z.$$

Furthermore, to satisfy the constraint $\sum_{x}\pi_{x}=1$, we obtain

$$\lambda =-\sum_{x,y}A\left(\delta \left(x,y\right)\right)m_{\beta }\left(x,y\right).$$

Therefore, we get

$$\sum_{x,y}A\left(\delta \left(x,y\right)\right)m_{\beta }\left(y,x\right)\left[\frac{I(X=z)}{\pi_{x}}-1\right]=0$$

and by making use of the fact that $\sum_{x,y}m_{\beta }\left(x,y\right)\left[\frac{I(X=z)}{\pi_{x}}-1\right]=0$, the above equation can be represented as

$$\sum_{x,y}w\left(\delta \left(x,y\right)\right)n_{x,y}\left[\frac{I(X=x)}{\pi_{x}}-1\right]=0$$

for any *x* where I(X=x) is the indicator function of the event {X=x}. □

### Appendix A.2. Proof of Proposition 5

Recall that $\beta_{\epsilon }$ is a solution of the set of estimating equation

(A1) $$\sum_{s,t}w\left(\delta_{\epsilon }\left(s,t\right)\right)u\left(t|s;\beta_{\epsilon }\right)d_{\epsilon }\left(s,t\right)=0,$$

where $d_{\epsilon }\left(s,t\right)=\left(1-\epsilon \right)d\left(s,t\right)+\epsilon \nabla_{x,y}\left(s,t\right)$ and $u\left(t|s;\beta \right)=\frac{\nabla_{\beta }m_{\beta }\left(s,t\right)}{m_{\beta }\left(s,t\right)}=\nabla_{\beta }\ln\left[m_{\beta }\left(s,t\right)\right]$ is a *p*-dimensional vector.

The influence function of β is calculated by differentiating, with respect to ϵ, the quantity (A1), and evaluating the derivative at ϵ=0. Thus, we need

(A2) $$\begin{array}{cc} \frac{d}{d\epsilon }\{ & \sum_{s,t}w\left(\delta_{\epsilon }\left(s,t\right)\right)u\left(t|s;\beta_{\epsilon }\right)d\left(s,t\right)\\ \; & -\epsilon \sum_{s,t}w\left(\delta_{\epsilon }\left(s,t\right)\right)u\left(t|s;\beta_{\epsilon }\right)d\left(s,t\right)\\ \; & +\epsilon \sum_{s,t}w\left(\delta_{\epsilon }\left(s,t\right)\right)u\left(t|s;\beta_{\epsilon }\right)\nabla_{\left(x,y\right)}\left(s,t\right)\}{|}_{\epsilon =0}=0. \end{array}$$

Taking into account that $\delta_{\epsilon }\left(s,t\right)=\frac{d_{\epsilon }\left(s,t\right)}{m_{\beta }\left(s,t\right)}-1=\frac{d_{\epsilon }\left(s,t\right)}{m_{\beta }\left(t|s\right)\pi_{s}}-1$, the aforementioned evaluation implies

(A3) $$\begin{array}{cc} \{ & \sum_{s,t}\left(\delta_{0}\left(t\right)+1\right)w_{0}^{\prime }\left(\delta_{0}\left(s,t\right)\right)u\left(t|s;\beta_{0}\right)u^{T}\left(t|s;\beta_{0}\right)d\left(s,t\right)\\ \; & -\sum_{s,t}w\left(\delta_{0}\left(s,t\right)\right)\nabla u\left(t|s;\beta_{0}\right)d\left(s,t\right)\}\beta_{0}^{\prime }\\ = & \sum_{s,t}\left\{\frac{I(s=x,y=t)}{m_{\beta_{0}}\left(t|s\right)\pi_{s}}-\frac{d(s,t)}{m_{\beta_{0}}\left(t|s\right)\pi_{s}}w^{\prime }\left(\delta_{0}\left(s,t\right)\right)\right\}u\left(t|s;\beta_{0}\right)d\left(s,t\right)\\ \; & -\sum_{s,t}w\left(\delta_{0}\left(s,t\right)\right)u\left(t|s;\beta_{0}\right)d\left(s,t\right)+w\left(\delta_{0}\left(x,y\right)\right)u\left(y|x;\beta_{0}\right), \end{array}$$

which implies that

$$\beta_{0}^{\prime }=IF\left(\beta ;F\right)={\left[A\left(d\right)\right]}^{-1}B\left(x,y;d\right).$$

### Appendix A.3. Assumptions of Theorem 1

The following assumptions are needed to be able to establish asymptotic normality of the estimators.

1. The weight functions are nonnegative, bounded and differentiable with respect to δ.
2. The weight function is regular, that is, $w^{\prime }\left(\delta \right)\left(\delta +1\right)$ is bounded, where $w^{\prime }\left(\delta \right)$ is the derivative of *w* with respect to δ.
3. $\sum_{x,y}m^{\frac{1}{2}}\left(x,y\right)E\left[u_{k}^{2}\left(y|x;\beta_{0}\right)\right]<\infty .$
4. The elements of the Fisher information matrix are finite and the Fisher information matrix is nonsingular.
5. $\sum_{x,y}m^{\frac{1}{2}}\left(x,y\right)E\left[u_{i}^{2}\left(y|x;\beta_{0}\right)u_{j}^{2}\left(y|x;\beta_{0}\right)\right]<\infty \;\;\forall i,j=1,2,\cdots ,p.$
6. If $\beta_{0}$ denotes the true value of β, there exist functions $M_{ijk}\left(x\right)$ such that $|u_{ijk}\left(y|x;\beta_{0}\right)|\leq M_{ijk}\left(x\right)$, ∀β with $\|\beta -\beta_{0}{\|}^{2}<r\left(\beta_{0}\right)$, $r(\beta_{0})<0$ and $E_{\beta_{0}}\left|M_{ijk}\left(y|x\right)\right|<\infty ,\;\;\forall i,j,k.$
7. If $\beta_{0}$ denotes the true value of β, there is a neighborhood $N(\beta_{0})$ such that for $\beta \in N(\beta_{0})$ the quantity $|u_{t}\left(y|x;\beta_{0}\right)u_{i}\left(y|x;\beta_{0}\right)u_{e}\left(y|x;\beta_{0}\right)|$ are bounded by $M_{1}\left(y|x\right)$ and $M_{2}\left(y|x\right)$ respectively, such that their corresponding expectations are finite.
8. $A^{\prime \prime }\left(\delta +1\right)\left(\delta +1\right)$ is bounded, where $A^{\prime \prime }$ denotes the second derivative of *A* with respect to δ.
